# Multilocus genotyping of *Giardia duodenalis* isolates from children in Oromia Special Zone, central Ethiopia

**DOI:** 10.1186/s12866-016-0706-7

**Published:** 2016-05-21

**Authors:** Teklu Wegayehu, Md Robiul Karim, Junqiang Li, Haileeyesus Adamu, Berhanu Erko, Longxian Zhang, Getachew Tilahun

**Affiliations:** Aklilu Lemma Institute of Pathobiology, Addis Ababa University, Addis Ababa, Ethiopia; College of Natural Sciences, Arba Minch University, Arba Minch, Ethiopia; Institute of Biotechnology, Addis Ababa University, Addis Ababa, Ethiopia; Zhongshan School of Medicine, Sun Yat-sen University, Guangzhou, China; College of Animal Sciences and Veterinary Medicine, Henan Agricultural University, Zhengzhou, Henan China

**Keywords:** *Giardia duodenalis*, Assemblages, Genetic diversity, Asymptomatic children, Ethiopia

## Abstract

**Background:**

*Giardia duodenalis* is the etiologic agent of giardiasis in humans and other mammals worldwide. The burden of disease is high among children in developing countries where sanitation is inadequate. However, the epidemiology and genetic diversity of this parasite is poorly understood in Ethiopia. This study aimed to determine the prevalence and genetic diversity of *G. duodenalis* in asymptomatic children in Oromia Special Zone, central Ethiopia.

**Results:**

A total of 286 fresh fecal specimens were collected from children and screened using microscopy and PCR. The prevalence of *Giardia duodenalis* was 10.8 % (31/286) and 16.8 % (48/286) as detected by microscopy and nested PCR, respectively. The infection rate by the study area, sex and age group difference was not significant (*P* > 0.05). Genotyping results showed that 22.9 % (11/48) of the isolates belonged to assemblage A while 77.1 % (37/48) belonged to assemblage B. Although double peaks were observed at the chromatogram level, no mixed assemblage or sub-assemblage infections were demonstrated. Isolates of assemblage A mostly belonged to the sub-assemblage AII and showed similarity with previously described isolates. However, there was great genetic variability within assemblage B that showed heterogeneous nucleotide positions. Fifteen of them were new genotypes: 5 at the triose phosphate isomerase *(tpi),* 2 at the β-giardin *(bg),* and 8 at the glutamate dehydrogenase *(gdh)* genes.

**Conclusions:**

*Giardia duodenalis* mainly assemblage B infection was predominant among the asymptomatic children in the study area. The high polymorphism found in isolates of assemblage B warrants a more defining tool to discriminate assemblage B at the sub-assemblage level. The findings of the present study indicate that there is a need to carry out national screening programs aiming to detect asymptomatic infections to minimize the reservoir of the disease.

**Electronic supplementary material:**

The online version of this article (doi:10.1186/s12866-016-0706-7) contains supplementary material, which is available to authorized users.

## Background

*G. duodenalis* (syn. *G. intestinalis*, *G. lamblia*) is frequently identified intestinal parasite that infects human and other mammals worldwide [1–3]. It is the etiologic agent of gastroenteritis known as giardiasis, and is included in the neglected disease initiative together with cryptosporidiosis by the World Health Organization in 2004 [4]. *G. duodenalis* has direct life cycle in which the parasite alternates between the cyst and the trophozoite stages [1]. The infective stage is transmitted through the fecal-oral route either by ingestion of contaminated food and water or directly from infected individuals [1, 5].

The public health impact of giardiasis is significant because of its tendency to cause major outbreaks and emergency responses, and because of its effects on growth and cognitive functions in children [3]. Clinical manifestations of the disease in human are quite variable, ranging from the asymptomatic to acute or chronic diarrhea, dehydration, abdominal pain, nausea, vomiting, and weight loss [6]. The severity of giardiasis is determined by the interaction between the host factors such as the developmental, nutritional and immunological status, and virulence factors of the parasite [7].

It is well documented that *G. duodenalis* represents a species complex and has the broadest host range [3, 4]. Molecular characterization and phylogenetic analysis have revealed at least eight major genetic groups (assemblages) of *G. duodenalis* that have different host ranges and specificities [3, 8]. Two of them (assemblages A and B) are found in both humans and animals. The remaining six assemblages (C to H) are host-specific (specific to animals). However, assemblages C, D, E, and F have been reported to cause human giardiasis in rare cases [3, 9, 10].

There is also a sub-structuring within assemblages A and B by sequence data from multiple loci into five sub-assemblages (named AI-III and BIII-IV), some of which may have zoonotic potential [3, 8]. A multilocus sequence typing approach and nomenclature based on the use of *bg*, *gdh* and *tpi* genes has been proposed for assemblage A [3]. However, other typing strategies may be needed for assemblage B because of its high genetic heterogeneity among isolates in most markers [3, 11].

Previous studies conducted in Ethiopia on *G. duodenalis* infection gave due attention to the prevalence and risk factors among different community groups using microscopy [12–19]. According to those epidemiological studies, the infection rate ranged from 2.0 to 35.3 % with the highest prevalence among children of 1-15 years [12, 13]. Although a number of studies have been conducted on the distribution and prevalence of this parasite in different parts of the country, none of these previous works had determined the assemblage and sub-assemblage diversity in humans living in close contact with cattle and their manure to assess the potential of zoonotic infections. In addition, very little information is available on molecular epidemiology of *G. duodenalis* in the country [20, 21]. Therefore, the objective of this study was to determine the prevalence and genetic diversity of *G. duodenalis* in children who had close contact with animals to assess the existence of zoonotic assemblages and sub-assemblages.

## Results

A total of 312 study participants were selected for this study. However, 26 (8.3 %) of the children were unable to provide the specimen and hence excluded. For this reason a total of 286 children were included, among which 154 (53.8 %) were males and 132 (46.2 %) were females with male to female ratio of 1:0.8. Furthermore, the children were stratified in two age groups: < 5 years making 130 (45.5 %) of the children and 5-14 years accounting for the rest 156 (54.5 %) children (Table 1).

**Table 1:** PCR based prevalence of *G. duodenalis* in children by study site, sex and age group in Oromia Special Zone, central Ethiopia (January - June, 2014)

Parameters	No. of samples examined	*G. duodenalis*		
		No. of samples positives n (%)	*χ*2	*P* value
Study site				
Holetta	102	15 (14.7)		
Sendafa	101	20 (19.8)	1.115	0.573
Chancho	83	13 (15.7)		
Sex				
Male	154	27 (17.5)	0.293	0.588
Female	132	21 (15.9)		
Age group				
< 5 years	130	21 (16.2)	0.011	0.917
5-14 years	156	27 (17.3)		

Key: *χ*2 and *P* values compare the prevalence between study sites, sex and age groups in children

### Prevalence of *G. duodenalis* infection

Microscopic analysis showed that the prevalence of *G. duodenalis* infection in children was 10.8 % (31/286). On the other hand, 16.8 % (48/286) of the DNA samples were PCR positive based on *tpi, bg* and *gdh* genes. Although the prevalence of *G. duodenalis* infection varied across study areas, the difference was not statistically significant (*P* > 0.05) (Table 1). Similarly, as noted from Table 1, the prevalence of infection by sex and age group did not show any significant difference.

### Assemblages and sub-assemblages of *G. duodenalis*

The *Giardia* assemblage was successfully determined from 48 specimens by DNA sequencing at *tpi, bg* and *gdh* markers. Sequence analysis showed that 22.9 % (11/48) isolates belonged to *G. duodenalis* assemblage A and 77.1 % (37/48) isolates displayed assemblage B. Although double peaks were observed at the chromatogram level, no mixed infections with assemblages or sub-asemblages of *G. duodenalis* were demonstrated. Out of the 11 *G. duodenalis* assemblages A recorded in children, six assemblages were identified by *tpi, bg* and *gdh* genes in common. The remaining five assemblages were identified by *tpi* and *bg* genes. Assemblages and sub-assemblages showing known and novel DNA sequences are shown in Table 2.

**Table 2:** Assemblages and sub-assemblages of *G. duodenalis* determined by the three genes

Study site	Isolate code	Children		Assemblages and sub-assemblages		
		Sex	Age	*tpi*	*bg*	*gdh*
Holetta	HH-02	F	5-14 years	B (B2)	B	B*
	HH-06	F	5-14 years	AII	AII	AII
	HH-13	M	<5 years	B*	B	–
	HH-25	M	5–14 years	AII	AII	–
	HH-34	M	5–14 years	B (B2)	B	–
	HH-49	M	5–14 years	B	B*	B*
	HH-55	M	5–14 years	–	A (A3)	–
	HH-57	M	<5 years	–	A (A3)	–
	HH-70	F	5–14 years	B (B2)	B (B3)	B*
	HH-71	M	<5 years	B*	B	B*
	HH-72	M	<5 years	B*	B (B3)	B*
	HH-78	M	5–14 years	B*	–	–
	HH-80	M	5–14 years	AII	–	–
	HH-84	F	<5 years	B (WB9)	B	B*
	HH-93	M	5–14 years	AII	AII	AII
Sendafa	SH-03	F	5–14 years	B (B2)	-	BIV
	SH-12	M	5–14 years	B (B2)	B*	B*
	SH-14	M	5–14 years	B	B*	B*
	SH-18	M	5–14 years	B	B (B3)	B*
	SH-20	M	<5 years	AII	AII	AII
	SH-33	F	5–14 years	AII	AII	AII
	SH-35	F	5–14 years	B (B2)	B (B3)	B*
	SH-38	M	5–14 years	AII	AII	–
	SH-43	F	<5 years	B (WB8)	B (B3)	B*
	SH-48	F	5–14 years	B	–	–
	SH-49	M	5–14 years	B (B2)	B*	B*
	SH-51	F	<5 years	B	–	–
	SH-54	M	5–14 years	B (B2)	B	B*
	SH-69	M	5–14 years	AII	AII	AII
	SH-74	M	5–14 years	B (B2)	–	–
	SH-75	F	5–14 years	B	B	B*
	SH-82	M	5–14 years	AII	AII	AII
	SH-83	F	5–14 years	B*	B	B*
	SH-92	M	5–14 years	B (WB9)	B	B*
	SH-93	F	5–14 years	B (B2)	B	B*
Chancho	CH-20	F	5–14 years	B (B2)	–	–
	CH-26	F	5–14 years	B (WB9)	B (B3)	B*
	CH-36	F	5–14 years	B*	B (B3)	–
	CH-37	F	5–14 years	B*	B	–
	CH–40	M	5–14 years	B*	B (B3)	–
	CH-44	M	5–14 years	B (B2)	B	B*
	CH-49	F	5–14 years	B*	–	–
	CH-55	M	<5 years	B (B2)	B	B*
	DH-07	F	5–14 years	–	B	B*
	DH-08	F	5–14 years	B (B2)	B	–
	DH-11	M	5–14 years	B (B2)	–	–
	DH-17	M	5–14 years	B	B	–
	DH-21	M	5–14 years	B (B2)	B (B3)	–

Key: Asterisks (*) indicate novel genotypes; hyphens (–) indicate PCR-negative results

The sub-assemblages B2, B3, WB9 and WB8 were based on the BLAST results

### Genetic characterization of *G. duodenalis*

Sequences were further analyzed and compared with homologous sequences at database to determine genetic polymorphism within assemblages of *G. duodenalis*. Among the 45 human isolates that were sequenced at *tpi* gene, 9 isolates were assemblage A sub-assemblage AII (Table 2). They displayed a 100 % similarity with sequences registered in the GenBank under accession number AY368157.

Thirty six sequences at *tpi* locus were identified as assemblage B and showed 9 different nucleotide sequences (Table 3). Among them 16 sequences were identical to sub-assemblage B2 (GU564280); 7 sequences were identical to human sub-assemblage in India (JF918519); 3 sequences identical to sub-assemblage WB9 (KJ888985); and 1 sequence was found to be identical to sub-assemblage WB8 from wastewater in the USA (AY368169). The remaining 9 isolates were reported here for the first time and named as EB1 (*n* = 3), EB2 (*n* = 2). EB3 (*n* = 2), EB4 (*n* = 1), and EB5 (*n* = 1) (E for Ethiopia). They showed 99 % similarity with GenBank accession numbers KJ888988, AY368167, JF918519, AY228628, FJ560565, JF918520 and AY368171, respectively. The single nucleotide polymorphisms (SNPs) and overlapping nucleotide are shown in Table 3.

**Table 3:** The genetic variants found within sub-assemblages of *G. duodenalis* assemblage B at the *tpi* gene

sub-assemblages	GenBank accession no	No. of isolates	Nucleotide at position*									
			25	51	77	148	151	154	184	196	415	490
BIV (Ref.)	AF069560		A	A	T	G	T	T	C	A	A	C
B2	KT948101	16	G	*	C	*	C	C	*	G	G	*
B	KT948103	7	G	*	C	A	C	C	*	G	G	*
MB9	KT948104	3	*	*	*	*	*	C		G	G	*
WB8	KT948109	1	*	*	*	*	*	*	*	*	G	*
EB1	KT948105	3	G	*	C	*	*	*	*	G	G	*
EB2	KT948106	2	G	*	C	*	C	C	T	G	G	*
EB3.	KT948107	2	*	*	*	*	*	C	*	G	G	*
EB4	KT948108	1	*	G	C	*	C	C	*	G	G	T
EB5	KT948110	1	*	*	Y	*	*	*	*	*	*	*

Key: Asterisks (*) indicate nucleotide identity with the reference sequence; Y: C/T indicates overlapping nucleotides

Nucleotide positions are numbered according to the reference (ref.) sub-assemblage BIV partial sequence (GenBank accession number AF069560), with the first nucleotide as position 20

Out of the 39 human isolates amplified and successfully sequenced from *bg* gene, 10 were identified as assemblage A (two different nucleotide sequences), and the other 29 were identified as assemblage B (six different nucleotides) (Table 4). Of the 10 *bg* nucleotide sequences identified as assemblage A, 8 sequences showed 100 % resemblance to sub-assemblage AII (KT182087) obtained from humans in Turkey. The remaining 2 sequences also showed 100 % similarity to human isolates found from India with accession number JF918488.

**Table 4:** The genetic variants found within sub-assemblages of *G. duodenalis* assemblage A and B at the *bg* gene

sub-assemblages	GenBank accession no	No. of isolates	Nucleotide at position*						
			165	183	309	324	415	423	519
Ass-A									
A3 Ref.	AY072724		T	A	G	A	T	C	A
AII	KT948085	8	*	*	*	*	*	*	*
A	KT948085	2	*	*	*	*	C	T	*
Ass-B									
B3 Ref.	AY072727		C	A	C	C	C	A	T
B3 like1	KT948083	13	*	*	Y	*	*	*	*
B3 like2	KT948084	9	*	*	*	*	*	*	*
EB6	KT948086	3	*	X	T	*	*	*	Y
B3 like3	KT948088	2	*	*	*	*	*	*	C
B3 like4	KT948089	1	T	*	T	*	*	*	*
EB7	KT948090	1	T	*	*	T	*	*	*

Key: Asterisks (*) indicate nucleotide identity with the reference sequence; X: A/G and Y: C/T indicates overlapping nucleotides

Nucleotide positions are numbered according to the reference (ref.) sub-assemblages with the first nucleotide as position 1

From the 29 isolates at the *bg* gene of assemblage B, 13 was sub-assemblages associated with clinical and environmental samples in Brazil (KF922976); 9 were identical to sub-assemblage B3 (AY072727); 2 were identical to sub-assemblages associated with a water-borne outbreak in Norway (DQ090529); and 1 was identical to sub-assemblage in Birzil (KF922989). BLAST result of the other sequences with sequences in GenBank database was not identical to known sequences of assemblages B, resulting in two novel genotypes, named: EB6 (*n* = 3) and EB7 (*n* = 1). The number of SNPs differs from the reference sequences and double peaks at the chromatogram level are shown in Table 4.

Among the 28 human isolates at the *gdh* gene, 6 were identified as assemblage A (sub-assemblage AII) (Table 2) and showed 100 % similarity to waterborne isolate in Canada (EF507666). Similarly, out of the 22 isolates of assemblage B at the same locus, 9 genotypes were identified. Only one sequence corresponded to the already described sub-assemblage BIV (GenBank accession number EF507654). The rest sequences form eight novel genotypes, named as: EB8 (*n* = 7), EB9 (*n* = 3), EB10 (*n* = 3), EB11 (*n* = 2), EB12 (*n* = 2), EB13 (*n* = 2), EB14 (*n* = 1) and EB15 (*n* = 1). Alignment analysis revealed that the isolates differ from reference sequence EF507654 by four to eight SNPs and exhibit many double peaks at the chromatogram profile (Table 5).

**Table 5:** The genetic variants found within sub-assemblages of *G. duodenalis* assemblage B at the *gdh* gene

sub-assemblages	GenBank accession no	No. of isolates	Nucleotide at position*																			
			282	288	354	375	381	414	447	471	534	558	579	615	642	653	657	660	669	702	756	768
BIV (Ref.)	EF507654		C	C	T	C	C	C	G	T	C	C	C	C	T	A	G	A	C	C	G	C
BIV	KT948098	1	*	*	*	*	*	*	*	*	*	*	*	*	*	*	*	*	*	*	*	*
EB8	KT948092	7	T	T	Y	T	*	*	X	*	*	*	*	*	*	*	*	G	*	*	A	*
EB9	KT948093	3	T	T	C	*	*	*	*	*	*	*	*	*	G	*	*	X	*	*	*	Y
EB10	KT948094	3	T	T	Y	*	*	*	X	*	*	Y	*	*	*	*	*	X	*	*	X	*
EB11	KT948095	2	T	T	C	*	*	*	*	*	*	*	*	*	*	*	*	*	*	*	X	*
EB12	KT948096	2	T	T	C	*	*	*	*	*	*	T	*	*	G	*	*	*	*	*	*	*
EB13	KT948097	2	T	T	*	*	T	*	*	C	*	*	*	*	*	*	*	*	T	T	A	*
EB14	KT948099	1	T	T	*	*	*	Y	*	*	*	*	Y	*	G	X	X	*	*	*	*	T
EB15	KT948100	1	T	T	*	*	Y	*	*	*	Y	*	*	Y	G	*	*	*	*	*	*	T

Key: Asterisks (*) indicate nucleotide identity with the reference sequence; X: A/G and Y: C/T indicate overlapping nucleotides

Nucleotide positions are numbered according to the reference (ref.) sub-assemblage BIV partial sequence (GenBank accession number EF507654), with the first nucleotide as position 1

### Phylogenetic analysis

**Fig. 1:**
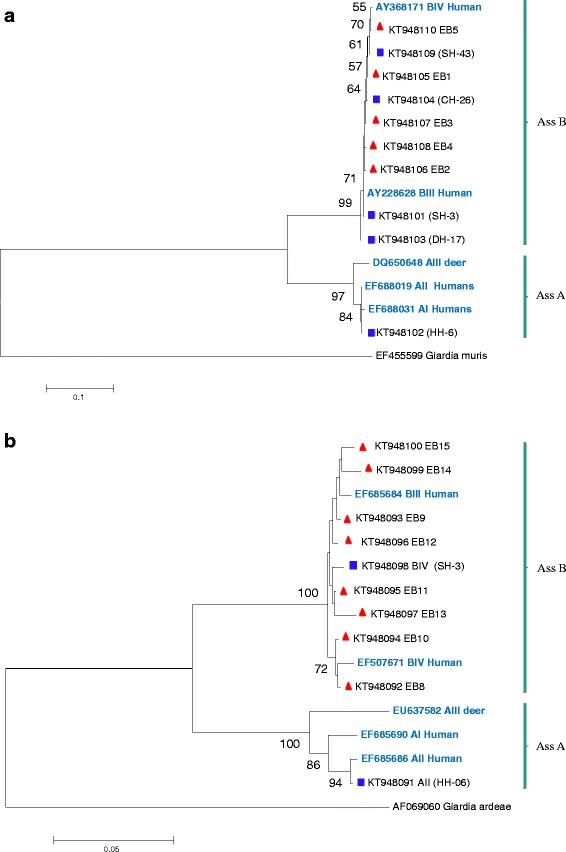
Phylogenetic tree of *G. duodenalis* based on nucleotide sequences of the *tpi* gene (**a**) and *gdh* gene (**b**). Trees were constructed using the neighbor-joining method based on genetic distance calculated by the Kimura 2-parameter model, implemented in MEGA version 5.2. Bootstrap values > 50 % from 1,000 replicates is shown on nodes. Reference sequences from the major *G. duodenalis* sub-assemblages are written by blue color in bold. Isolates showed known and novel sequences obtained from this study are marked by rectangles (blue) and triangles (red), respectively

The phylogenetic analysis of *G. duodenalis* sequence dataset obtained herein and those available in GenBank were concordant in revealing the existence of different clades for the *tpi* and *gdh* genes with in assemblages A and B. The novel genotypes at *tpi* gene, EB1 and EB5 were closely clustered with sub-assemblage BIV; but EB2, EB3 and EB4 were clustered with BIII (Fig. 1a). On the other hand, the novel genotypes at *gdh*: EB8 and EB10 were clustered with the BIV (accession number EF507671) whereas the others were clustered with BIII (Fig. 1b).

### Intestinal parasites other than *G. duodenalis*

Four intestinal parasites other than *G. duodenalis* were encountered in the children. The prevalence of *Entamoeba histolytica/dispar* was the highest being 8.7 % (25/286), followed by *Ascaris lumbricoides* 4.2 % (12/286), *Hymenolepis nana* 4.2 % (12/286) and *Enterobius vermicularis* 2.1 % (6/286).

## Discussion

The present study determines the prevalence and genetic diversity of *G. duodenalis* among children using multilocus genotyping in three districts in Oromia Special Zone, central Ethiopia. Fecal samples collected from children were screened by both microscopy and nested PCR. Although microscopy and PCR had similar sensitivities in a Dutch study [22], higher infection rate was recorded by the PCR in the present study. The superior sensitivity of PCR in detecting *G. duodenalis* has been shown in Danish patients [23] and in Rwandan children [24]. Hence, repeated microscopic examinations or the use of other diagnostic tools such as immunoassays could have reduced the discrepancy between PCR and microscopy in clinical setup.

In the present study, the microscopy based prevalence of *G. duodenalis* infection reported in children was 10.8 %. This was in agreement with previous reports 10.6 % [15], 13.8 % [16], and 11.7 % [18] conducted using similar technique in Ethiopia. On the other hand, the present finding was lower than the prevalence (15.0 %) reported by Ramos et al. [19] among patients with diarrhea in southern Ethiopia; 23.0 % by King et al. [17] from inhabitants in South Gondar; 26.6 % by Tigabu et al. [14] from children in selected village of Pawi Special District in Benishangul-Gumuz; and 35.3 % by Ayalew et al. [13] from children in Lege Dini. These variations could be due to difference in the hygiene practices, quality of drinking water, difference in environmental conditions of the study localities, and parental socio-economic status of the participants of these studies.

To the best of our knowledge, there were only two molecular studies on giardiasis in Ethiopia [20, 21]. These studies were presented the genetic diversity of assemblages and sub-assemblages of *G. duodenalis* from human fecal samples with confirmed giardiasis. The present study showed the PCR based prevalence of *Giardia* infection with all the three genes, and the overall prevalence was found to be 16.8 %. Although significant difference was not recorded in the overall prevalence among the study areas, *G. duodenalis* infection in children was slightly higher in Sendafa (20.0 %). This could be attributed to the similarity of quality of drinking water source, personal hygiene and environmental sanitary conditions observed in the study areas. The difference in prevalence of infection by sex and age group was also not significant among the children.

As to global distribution of assemblages A and B among humans, assemblage B is more prevalent than assemblage A as reviewed by Feng and Xiao, and Caccio and Ryan [3, 5]. The finding of the present study in children was in agreement with this global incidence that assemblage B predominates (77.1 %) assemblage A (22.9 %). Recently several studies showed similar pattern of distribution [21, 24–27]. However, the previous study conducted in symptomatic individuals in Ethiopia showed 52 % as assemblage A and 22 % as assemblage B [20]. Similar pattern of distribution of assemblages was also observed in hospitalized based studies conducted in Egypt [28]. Another molecular information from Spanish pediatric populations have shown that, in patients less than 5 years old, symptomatic giardiasis was present in 81.2 % of assemblage AII infections but present only in 34.6 % of assemblage B cases [29]. These variations in the infection rates of assemblage A and B might be associated with the clinical symptoms of the disease.

Isolates of assemblage A at the sub-assemblage level was successfully characterized at the three markers (Table 2). Regardless of the gene analyzed, all the sequences matched those of previously described isolates. No sequences showed novel polymorphisms. Sequences with heterogeneous positions were observed only in a small proportion of isolates at the *bg* locus. Most of the assemblage A isolates were assigned to the sub-assemblage AII consistently across the three loci, confirming the preponderance of this sub-assemblage in humans compared to sub-assemblage AI as previously shown in other studies using multi-locus sequence typing [11, 27, 30, 31].

The molecular analysis of assemblage B isolates was complicated by the occurrence of sequences with heterogeneous positions, with overlapping nucleotides observed across the three loci. This is consistent with the previous reports from different parts of the world [11, 27, 31]. A recent study conducted by Flecha et al. [21] in Gambo Hospital, Oromia Region, southern Ethiopia also showed an elevated genetic polymorphism in assemblage B using *gdh* and *bg* genes. The potential mechanisms to explain this feature are meiotic recombination [32] or mixed infection with assemblages or sub-assemblages of *G. duodenalis* [33]. In this study, most of the SNPs reported across the three loci exhibited clear chromatogram readings in both forward and reverse directions, although a number of double peaks were also detected, particularly at the *gdh* locus. However, sequence alignment analyses do not seem to support the presence of mixed infection with assemblages or sub-assemblages. Hence, the occurrence of heterogeneous sequences may be due to the presence of two nuclei, which are thought to accumulate mutations and evolve separately lead to allelic sequence heterozygosity.

The assignment of isolates of assemblages B to a particular sub-assemblage was not simple due to discrepancies between the different markers. At the *tpi* locus the majority of the isolates belonged to sub-assemblages that were part of the previously identified B2 group. The B2 group clustered nearby the sub-assemblage BIII by phylogenetic analysis, but whether it represents an actual sub-assemblage other than BIII and BIV has to be verified. Conversely, the analysis of the *bg* marker assigned some of isolates to the B3 but some of them are still not assigned to sub-assemblage due to a lack of information in GenBank database. On the other hand, the analysis of the *gdh* locus assigned one isolate to sub-assemblage BIV and all the others as novel genotypes.

The genotypic diversity observed in assemblage B isolates was much higher than the one observed in assemblage A. Among the five novel genotypes that were successfully amplified at the *tpi* locus, 3 genotypes (EB2, EB3 and EB4) were assigned to sub-assemblage BIII and 2 (EB1, and EB5) were assigned to sub-assemblage BIV. Similarly, among the eight novel genotypes that were successfully amplified at the *gdh* locus, EB8 and EB10 were assigned to sub-assemblage BIV, whereas the others were assigned to BIII (Fig. 1). Similar level of diversity at the three markers were reported in assemblage B parasites in patients from Sweden, German and England [27, 31, 34]. This observation was also consistent with the results obtained by molecular characterization of human isolates of *G. duodenalis* in previous studies in Ethiopia [20, 21]. There is still a lack of information about the level of genetic differentiation within *Giardia* assemblage B, and the classification in sub-assemblages can be complicated by the use of an imprecise terminology in naming parasite isolates [8].

Much of the available data on the correlation between clinical presentation and assemblages of *G. duodenalis* is inconsistent [20, 24, 29, 35–41]. Finding of previous studies in Ethiopia [20, 21], Netherlands [35] and Argentina [41] have shown that symptomatic infection was more associated with assemblage B. In contrast, other studies in Australia [36], Bangladesh [39], and Ruwanda [24] reported that assemblage A was more likely to be found in symptomatic children with diarrhea while assemblage B was more prevalent in asymptomatic children. Although the correlation was not assessed in this study, the higher infection rate of assemblage B found in asymptomatic children might support the observations that assemblage B was predominantly found in infected subjects without clinical manifestations.

## Conclusion

Our data provide evidence of a high prevalence and genetic diversity of *G. duodenalis* assemblage B in asymptomatic children in Oromia Soecial Zone, central Ethiopia with majority of them being novel genotypes. Measures including health education on personal and environmental hygiene, and national screening programs aiming to detect asymptomatic infections would help to minimize the reservoir of the disease. Moreover, further studies are needed to demonstrate the potential correlation between assemblages of *G. duodenalis* and clinical features in infected individuals.

## Methods

### Study area

Community-based cross-sectional study was conducted between January and June 2014 in Holetta, Sendafa and Chancho and their surroundings of Oromia Special Zone, central Ethiopia. The three study areas are located at a distance of ~40 km west, northeast and north of the capital city, Addis Ababa. Based on the available climatological data, the mean annual rainfall of the Special Zone varies from 700 mm to 1400 mm in lowlands and highlands, respectively. The mean annual temperature of the Zone ranges between 20 to 25 °C in the lowlands and 10 to15 °C in the highlands. Mixed farming is the major livelihood of the people in the area. The owned livestock includes cattle, sheep and poultry. Risk factors that might affect the prevalence of intestinal parasites, such as source of drinking water, personal hygiene, environmental sanitation, and contact with animals and their manure were comparable in the study areas.

### Sampling techniques

In view of the objectives of the study, children were recruited based on the following criteria.

**Inclusion criteria:** Parents/guardians of children having domestic animals at their home and consented to participate in the study were included. **Exclusion criteria:** Infants with ages younger than 1 year; new settlers (stayed less than 3 weeks in the area); children who had no close contact with domestic animals and their manure were excluded. Thus, a total of 286 children age younger than 14 years (102 from Holetta, 101 from Sendafa and 83 from Chancho) were included in the study.

### Specimen collection and microscopy

Single fresh fecal sample was collected from each consenting study children in a labeled and sterile fecal container. The information concerning socio-demographic characteristics of the study participants (sex and age), and contact with animals and their manure were taken during the sample collection. A portion of each specimen was examined under light microscope to detect cysts of *G. duodenalis* and other intestinal parasites using the Lugol’s iodine staining at 10X and 40X magnifications. Some diarrheic stools were also examined by direct wet mount with saline (0.85 % sodium chloride solution) to observe trophozoites and motile intestinal parasites. The remaining stool was preserved in 2.5 % potassium dichromate and transported to ALIPB at ambient temperature and stored at 4 °C prior to DNA extraction.

### DNA extraction

The preserved fecal specimens were washed with deionized water until the potassium dichromate was removed. Genomic DNA was extracted from each fecal sample using the E.Z.N.A.® Stool DNA kit (Omega Biotek Inc., Norcross, USA). Briefly, about 50-100 mg of stool sample was added in a 2 ml centrifuge tube containing 200 mg of glass beads and placed on ice. Following, 300 μl buffer SP1 and proteinase K were added, and incubated at 70 °C for 10 min. Subsequently, all the procedures outlined in product manual were performed according to the manufacturer’s protocol. Finally, DNA was eluted in 200 μl of elution buffer and the extract was stored at -20 °C until PCR.

### Nested PCR

All extracted DNA samples were tested for *G. duodenalis* using nested PCR amplification of the *tpi, bg* and *gdh* genes. The nested PCR using previously described mixes and PCR conditions were used to amplify fragments of the *bg* gene [42], the *gdh* gene [43] and the *tpi* gene [44], with some modifications. The primers and annealing temperature used were similar to the one used by Wang et al. [45] (Additional file 1). The PCR reactions were conducted in 25 μl reaction mixtures for the *tpi* and *gdh* loci, containing 1× PCR buffer (TaKaRa Shuzo Co., Ltd., Otsu, Japan), 200 μM each dNTP (TaKaRa Shuzo Co., Ltd.), 0.4 μM each primer, 1 unit of rTaq DNA polymerase (TaKaRa Shuzo Co., Ltd.), and 2 μL of DNA sample. In the *bg* protocol, 1X Ex Taq buffer (TaKaRa Shuzo Co., Ltd.) and Ex Taq DNA polymerase (TaKaRa Shuzo Co., Ltd.) were used instead of 1X PCR buffer and rTaq. The secondary PCR reactions were similar to the primary PCR with the exception that 2 μl of the primary PCR product was used as a template. In addition, the annealing temperature of *bg* gene was lowered from 65 to 55 °C. Both positive and negative controls were included in each round of PCR to validate results. The amplified products were separated by electrophoresis on 1 % agarose gel stained with ethidium bromide and visualized under UV trans-illuminator. The PCR was conducted at the International Joint Research Laboratory for Zoonotic Diseases at Henan Agricultural University, China.

### DNA sequencing and analysis

All positive PCR products were purified using Montage PCR filters (Millipore, Bedford, MA) and sequenced using an ABI BigDye Terminator v. 3.1 cycle sequencing kit (Applied Biosystems, Foster City, CA) on an ABI 3100 automated sequencer (Applied Biosystems). Sequence accuracy was confirmed by sequencing both directions with primers used for the secondary PCRs. The raw nucleotide sequences and chromatograms of both forward and reverse directions were viewed using the EditSeq 5.0 and Chromas 2.4 program, respectively. The presence of double peaks at the chromatogram level was verified and the sequences were aligned and analyzed using ClustalX software. Consensus sequences were then compared to homologous sequences in GenBank using the Basic Local Alignment Search Tool (BLAST) (http://www.ncbi.nlm.nih.gov/blast/) to determine the *G. duodenalis* assemblages and sub-assemblages.

### Phylogenetic analysis

A neighbor-joining tree was constructed using the Molecular Evolutionary Genetics Analysis (MEGA) program version 4.0 to estimate the evolutionary distance, based on genetic distance calculated by Kimura-2-parameter model. The reliability of the phylogenetic tree groupings was assessed by bootstrap analysis with 1000 replicates.

### Statistical analysis

Data were computerized using EpiData version 3.1 and imported to STATA Software for analysis. Chi square test was used to verify possible association of *G. duodenalis* infection with sex, age and study areas. Values were considered to be statistically significant when the *P*-value was < 0.05.

### Ethics, approval and consent to participate

Ethical approval was obtained from the Institutional Review Board of Aklilu Lemma Institute of Pathobiology (ALIPB), Addis Ababa University and the National Health Research Ethics Review Committee. Support letters were obtained from Oromia Special Zone Health Office and health administrative at community level. The objectives of the study were explained to parents or guardians of the selected children before the collection of the specimens and written consent was obtained. Study participants found positive for giardiasis and other intestinal parasites were treated using appropriate drugs administered by physicians at the health center.

### Availability of data and materials

The dataset supporting the conclusions of this article is available in the GenBank database repository under the accession numbers: KT948101 to KT948110 and KT948083 to KT948100.

## Additional file

Additional file 1: Summary of the target genes, primers and annealing temperature of *G. duodenalis* used in this study. (DOCX 15 kb)

## Abbreviations

ALIPB: Aklilu Lemma Institute of Pathobiology
*bg*: β-giardin
*gdh*: glutamate dehydrogenase
SNPs: single nucleotide polymorphisms
*tpi*: triose phosphate isomerase
