# Supplementary material for: Multilocus genotyping of Giardia duodenalis isolates from children in Oromia Special Zone, central Ethiopia
Source: BMC Microbiol. 2016 May 21;16:89. doi: 10.1186/s12866-016-0706-7 (PMC4875739; doi:10.1186/s12866-016-0706-7)
Supplement: Additional file 1: — Summary of the target genes, primers and annealing temperature of G. duodenalis used in this study. (DOCX 15 kb) [file 12866_2016_706_MOESM1_ESM.docx]

Additional file 1 Summary of the target genes, primers and annealing temperature of *G. duodenalis* used in this study

| **Pathogen/Gene** | **Primer (sequence 5´-3´)** | **Annealing**  **temp (^0^C)** | **length (bp)** |
| --- | --- | --- | --- |
| ***tpi*** | AL3543 (AAATIATGCCTGCTCGTCG) | 50 | 605 |
|  | AL3546 (CAAACCTTITCCGCAAACC) |  |  |
|  | AL3544 (CCCTTCATCGGIGGTAACTT) | 50 | 530 |
|  | AL3545 (GTGGCCACCACICCCGTGCC) |  |  |
| ***bg*** | G7 (AAGCCCGACGACCTCACCCGCAGTGC) | 65 | 753 |
|  | G759 (GAGGCCGCCCTGGATCTTCGAGACGAC) |  |  |
|  | BG-F2 (GAACGAACGAGATCGAGGTCCG) | 55 | 511 |
|  | BG-R2 (CTCGACGAGCTTCGTGTT) |  |  |
| ***gdh*** | Ghd1 (TTCCGTRTYCAGTACAACTC) | 50 | - |
|  | Gdh2 (ACCTCGTTCTGRGTGGCGCA) |  |  |
|  | Gdh3 (ATGACYGAGCTYCAGAGGCACGT) | 50 | 520 |
|  | Gdh4 (GTGGCGCARGGCATGATGCA) |  |  |

Adapted from Wang *et al.*, 2014
